# Investigation on the Optical Properties of Micro-LEDs Based on InGaN Quantum Dots Grown by Molecular Beam Epitaxy

**DOI:** 10.3390/nano13081346

**Published:** 2023-04-12

**Authors:** Ying Gu, Yi Gong, Peng Zhang, Haowen Hua, Shan Jin, Wenxian Yang, Jianjun Zhu, Shulong Lu

**Affiliations:** 1 School of Nano-Tech and Nano-Bionics, University of Science and Technology of China, Hefei 230026, China; ygu2021@sinano.ac.cn (Y.G.); hwhua2022@sinano.ac.cn (H.H.); 2 Key Lab of Nanodevices and Applications, Suzhou Institute of Nano-Tech and Nano-Bionics (SINANO), Chinese Academy of Sciences (CAS), Suzhou 215123, China; ygong2022@sinano.ac.cn (Y.G.); sjin2017@sinano.ac.cn (S.J.); sllu2008@sinano.ac.cn (S.L.); 3 School of Physical Science and Technology, Inner Mongolia University, Hohhot 010021, China

**Keywords:** InGaN quantum dots, micro-LEDs, molecular beam epitaxy, size effect

## Abstract

InGaN quantum dots (QDs) have attracted significant attention as a promising material for high-efficiency micro-LEDs. In this study, plasma-assisted molecular beam epitaxy (PA-MBE) was used to grow self-assembled InGaN QDs for the fabrication of green micro-LEDs. The InGaN QDs exhibited a high density of over 3.0 × 10^10^ cm^−2^, along with good dispersion and uniform size distribution. Micro-LEDs based on QDs with side lengths of the square mesa of 4, 8, 10, and 20 μm were prepared. Attributed to the shielding effect of QDs on the polarized field, luminescence tests indicated that InGaN QDs micro-LEDs exhibited excellent wavelength stability with increasing injection current density. The micro-LEDs with a side length of 8 μm showed a shift of 16.9 nm in the peak of emission wavelength as the injection current increased from 1 A/cm^2^ to 1000 A/cm^2^. Furthermore, InGaN QDs micro-LEDs maintained good performance stability with decreasing platform size at low current density. The EQE peak of the 8 μm micro-LEDs is 0.42%, which is 91% of the EQE peak of the 20 µm devices. This phenomenon can be attributed to the confinement effect of QDs on carriers, which is significant for the development of full-color micro-LED displays.

## 1. Introduction

Micro-LEDs refer to miniaturized light-emitting devices based on traditional LEDs, with the diameter of the emission surface generally below 100 µm [1]. Combining multiple independent micro-LEDs into a display device can achieve high brightness, high dynamic range, high contrast, and ultra-long lifespan display effects [2,3,4]. The structure of micro-LED display devices is simple and does not require a backlight source [5], thus, it has lower power consumption and higher brightness. In addition, micro-LED display devices have extremely high contrast and dynamic range, thus, high-quality image and video displays can be achieved. Micro-LED display technology has wide application prospects in fields such as smartphones, automotive displays, projectors, and display walls, and is one of the important development directions for future display technology [6,7,8]. Furthermore, GaN-based micro-LEDs were first reported in 2000, that is, S.X. Jin fabricated micro-LED devices with a diameter of 12 µm and an electrode-interconnected micro-LED array [9,10]. In recent years, GaN-based micro-LEDs have been considered a promising candidate for the next generation display due to outstanding advantages including high efficiency, high resolution, and long lifetime [11,12]. However, when used in display technology, such as near-eye display (NED) technology, GaN-based devices always show a low external quantum efficiency (EQE) under the working current density, which is often lower than 2 A cm^−2^ [13]. This phenomenon is mainly caused by poor crystal quality and the quantum confined Stark effect (QCSE) arising from the strong polarization-induced electrical field in the high In-content InGaN/GaN multi-quantum wells (MQWs) active region. In addition, the need for high wavelength stability is consistently increasing [14]. However, the emission wavelength of GaN-based micro-LEDs consistently exhibits a blueshift in response to increasing injection current. Additionally, the “size effect” of micro-LEDs has become an important factor limiting their development towards higher resolutions [15,16]. This effect describes the phenomenon of device performance deteriorating as the size of micro-LEDs decreases.

Compared with InGaN quantum well (QW) materials, InGaN QDs have advantages as active region materials for LEDs and micro-LEDs in many aspects [17]. Self-assembled InGaN QDs have weaker QCSE by suppressing the spatial separation of electrons and holes, and hence improve the internal quantum efficiency of QDs-based optical devices [18,19]. In addition, InGaN QDs have a strong quantum confinement effect. In the last three decades, LEDs and micro-LEDs based on InGaN quantum dots (QDs) have been studied by many groups [20,21,22,23]. For example, Chunyu Zhao reported the built-in fields in the QDs were effectively screened, resulting in improved performance of the QD LEDs [24]. As a result, these green QD LEDs exhibit high-temperature stability and operate with minimal efficiency droop at a current density of up to 106 A/cm^2^. Lei Wang reported that in comparison to QW micro-LEDs, QDs grown in Stranski–Krastanov (S-K) mode can shift the efficiency peak of a micro-LED to an incredibly low current density of 0.5 A cm^−2^ while achieving an EQE of 18.2% [25]. However, there is still a lack of systematic research and reports on the performance of different sizes of micro-LEDs based on InGaN QDs grown by MBE.

Herein, this paper focuses on the fabrication of green micro-LEDs based on self-assembled InGaN QDs grown by molecular beam epitaxy (MBE) and the investigation of their optical properties. Atomic force microscopy (AFM) and spherical aberration-corrected transmission electron microscope (AC-TEM) tests were used to study the surface morphology and microstructure of InGaN QDs materials. The EL method was used to measure the EQE, EL spectrum, and peak wavelength of micro-LEDs, and electrical testing was conducted to obtain the I-V curve. InGaN QDs-based micro-LEDs, exhibiting excellent wavelength stability with injection current increasing form to 1 A/cm^2^ to 1000 A/cm^2^, have been fabricated in different sizes. As the size decreases, the micro-LEDs do not exhibit a significant decline in efficiency.

## 2. Experiment Details

### 2.1. Growth of InGaN QD-Based LED Wafers

The GaN templates were grown on sapphire wafers with a cut-off angle toward the m-axis of 0.2° by metalorganic chemical vapor deposition (MOCVD). After that, the InGaN quantum dots (QDs) sample and InGaN QD-based micro-LED wafer were grown by a Veeco Gen20A plasma-assisted molecular beam epitaxy (PA-MBE) system. Before loading the samples into the growth chamber, solvent cleaning and thermal cleaning were performed. Thermal cleaning is a process of removing surface contaminants and oxide film for 1 h at 200 °C in a Load-lock chamber and 0.5 h at 760 °C in a growth chamber. The growth modes were monitored by reflection high energy electron diffraction (RHEED) in situ. The growth temperature was monitored by a BandiT system.

**Figure 1:**
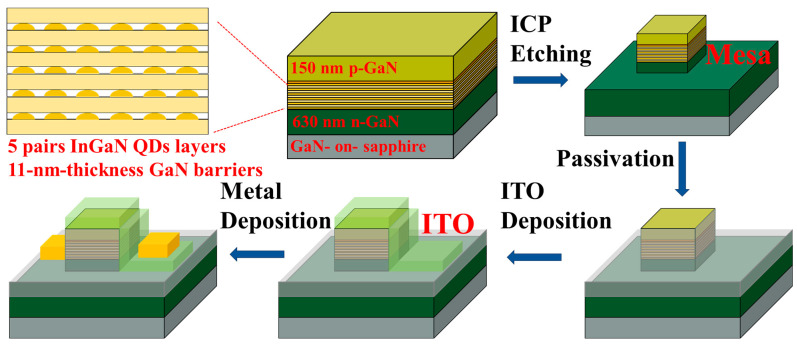
Schematic diagram of the QD micro-LED structure and the processing flowcharts of the QD micro-LED.

Figure 1 shows the schematic of the micro-LED structure using InGaN/GaN MQDs as an active region, including 5 pairs of InGaN QDs layers and 11-nm-thickness GaN barriers. The structure consists of a 630 nm Si-doped n-GaN layer, the MQDs structure, a 13.4 nm AlGaN electrical barrier layer (EBL), and a 150 nm Mg-doped p-GaN layer. InGaN QDs were grown at 605 °C with In flux in beam equivalent pressure (BEP) of 5.8 × 10^−9^ Torr and Ga flux of 3.6 × 10^−9^ Torr, followed by a GaN barrier layer grown at a low temperature of 650 °C with Ga flux of 5.2 × 10^−8^ Torr to reduce the damage to QDs active region.

### 2.2. Device Fabrication

The structure and the device fabrication process diagram of the QD-based micro-LEDs are illustrated in Figure 1. The first step is inductively coupled plasma (ICP) etching to form the micro-LED square mesa, and the side lengths were 4, 8, 10, and 20 µm, respectively. Then, 20 nm Al_2_O_3_ and 200 nm SiO_2_ were deposited as the passivation layer by atomic layer deposition (ALD) and plasma-enhanced chemical vapor deposition (PECVD), respectively. A dry etching process was carried out to form a metal contact area for ITO and electrodes. After that, a 130 nm thick indium tin oxide (ITO) film was deposited as a current spreading layer by electron beam deposition. To form an ohmic contact, rapid thermal annealing was carried out at 550 °C in an N_2_ atmosphere. Lastly, Ti/Al/Ni/Au (200/1200/600/1200 Å) was deposited as the electrodes on the n-GaN and ITO layer. Single micro-LED chip and 10 × 10 micro-LED arrays were fabricated.

### 2.3. Measurements of Micromorphology and Optical Properties

Bruker Dimension ICON AFM was used to measure the surface morphology of InGaN QDs, and the Thermo Scientific Themis Z AC-TEM system was used for microstructure characterizations. The emission wavelength was determined by the electroluminescence (*EL*) method with an iHR 320 spectrometer. The light output power (*LOP*) was measured by a photometric integrating sphere, based on which, the external quantum efficiency (*EQE*) is calculated by the following:

(1) $$EQE=\frac{P_{LOP}/h\cdot \nu }{I/q}$$

where *I* is the injection current, *h* is Planck’s constant, *ν* is the light frequency, and *q* is the electron charge. A Keysight 4200 semiconductor parameter analyzer was used for direct current characterization.

## 3. Results and Discussion

### 3.1. Morphology and Optical Properties of InGaN QDs

**Figure 2:**
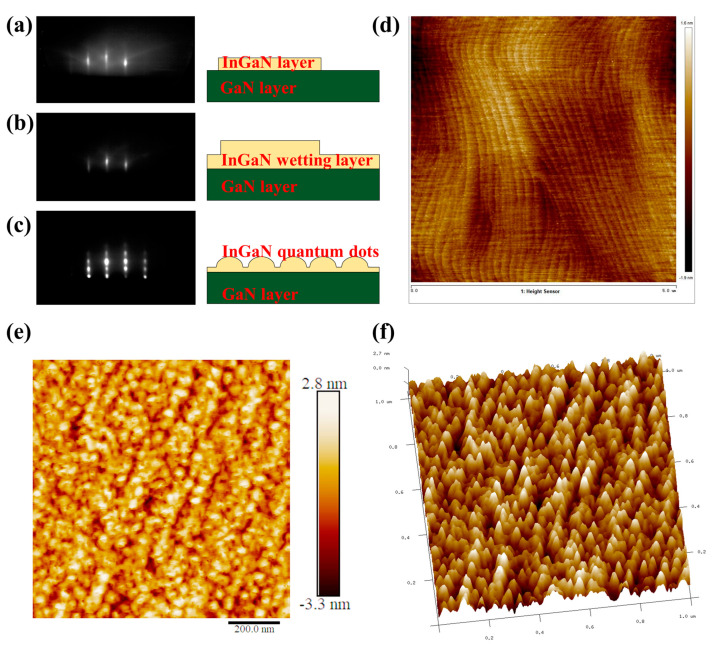
(**a**–**c**) The RHEED patterns of self-assembled InGaN QDs in different growth states; (**d**) the AFM image of GaN layer; (**e**,**f**) the 2D and 3DAFM images of InGaN QDs.

The growth modes of InGaN QDs by MBE were monitored by RHEED in situ. The RHEED patterns at different growing stages reveal the transition of surface morphology and microstructure. It can be found that the InGaN wetting layer was first formed in two-dimensional (2D) growth mode at the initial stage, as a streaky RHEED pattern was observed, as shown in Figure 2a,b. After growing a certain thickness of the InGaN wetting layer, RHEED patterns changed from streaky to spotty patterns in Figure 2c, which implied a spontaneous microstructure transition from 2D to 3D due to elastic relaxation, thus resulting in the formation of self-assembled InGaN QDs. Figure 2d is the AFM image of the GaN layer, and the atomic steps’ morphology can be observed. The 2D and 3D AFM images of InGaN QDs are presented in Figure 2e,f, respectively. Self-assembled InGaN QDs with truncated pyramidal features were obtained and uniformly distributed with a density greater than 3.0 × 10^10^ cm^−2^. The average height and diameter of dot sizes are mainly concentrated in about 1.6 nm and 31.7 nm, respectively. Furthermore, it can be observed that InGaN QDs were distributed along the surface steps of the GaN layer, due to the existence of the Ehrlich–Schwoebel barrier located at the step-edges, which will be studied in future work.

**Figure 3:**
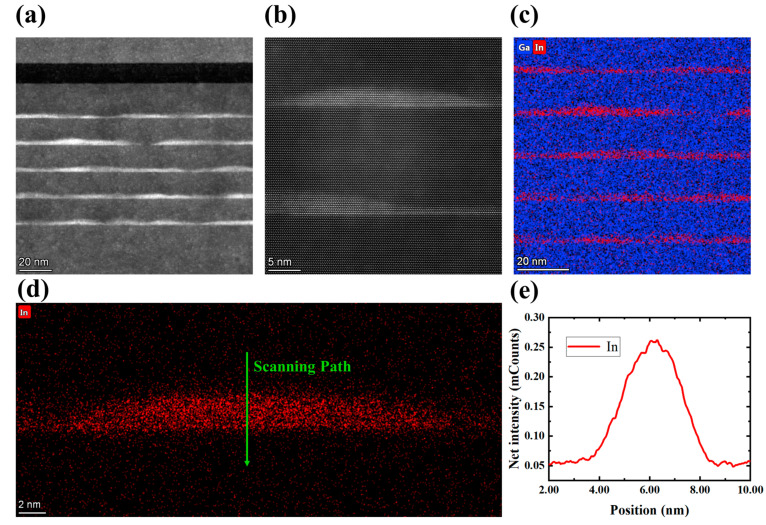
(**a**) Cross-sectional TEM image of InGaN/GaN MQDs in LED wafer; (**b**) enlarged local TEM image of InGaN QDs; (**c**,**d**) EDS mapping image of Ga and In element; (**e**) In element line scanning of the InGaN QD along the green arrow line.

From the cross-sectional TEM images of the LED wafer in Figure 3a and the enlarged TEM image of QD in Figure 3b, the InGaN/GaN MQDs structure with five cycles can be clearly observed. A clear and steep heterointerface between InGaN QDs and GaN barriers was formed. The energy dispersive spectroscopy (EDS) mapping test was used to investigate the spatial distribution of In and Ga elements, and the image is shown in Figure 3c. The result indicates that In elements are concentrated almost entirely in QDs. This phenomenon is a result of the “lattice pulling effect” happening during the growth of InGaN layers [26,27], causing a tendency for In atoms to segregate into the more relaxed regions, resulting in minimized system strain energy. The large lattice mismatch between InGaN and GaN will cause elastic strain existing in the InGaN layer, which acts as the major driving force for In surface segregation. Taking into account the three-dimensional island structure of InGaN QDs, the internal region of InGaN is expected to be more strain-relaxed compared with the surrounding regions. Therefore, In atoms are more likely to merge into InGaN QDs.

Furthermore, the line scanning in direction of the green arrow in Figure 3d was performed to study the In element distribution inside of the InGaN quantum island. According to the line scanning curve of In elements as shown in Figure 3e, the distribution of In elements inside the QDs is likely to be not uniform. There are two possible reasons for this phenomenon. The first one is the increasing of growth temperature after the growth of InGaN QDs from 605 to 650 °C to grow the GaN QB layer. The heating process will cause the desorption of some In atoms, resulting in a lower In component on the surface of QDs than the center part. The second one is the influence of the projection effect during the EDS measurement. Taking into account the 3D morphology of InGaN QDs, the truncated pyramidal shape is expected to bias the simple projected EDS profile, because the penetration range of the electron beam used for detection is deeper than the diameter of a QD. In this case, further study is needed to explore the spatial distribution and change mechanism of the In element in the interior of InGaN QD.

### 3.2. Optical Properties of InGaN QD Based Micro-LEDs

**Figure 4:**
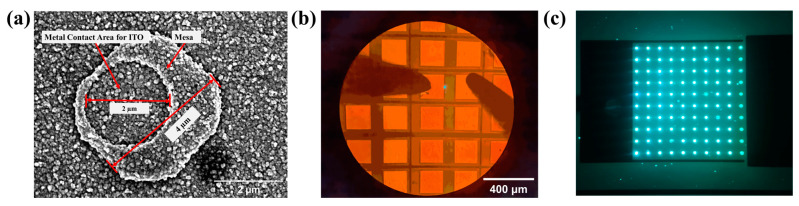
(**a**) SEM image of a 4 µm micro-LED; (**b**) the photo of a 4 µm micro-LED illuminated; (**c**) a photo of an array of 4 µm micro-LED illuminated.

Mesa sizes of the micro-LEDs were 4, 8, 10, and 20 µm, respectively. Figure 4a shows an SEM image of the micro-LED with a mesa size of 4 µm, where the passive layer window with a diameter of 2 µm is located on top of the mesa. Figure 4b shows a 4 µm micro-LED illuminated (the small green dot) on the probe station. Figure 4c shows an array of 4 µm micro-LEDs.

**Figure 5:**
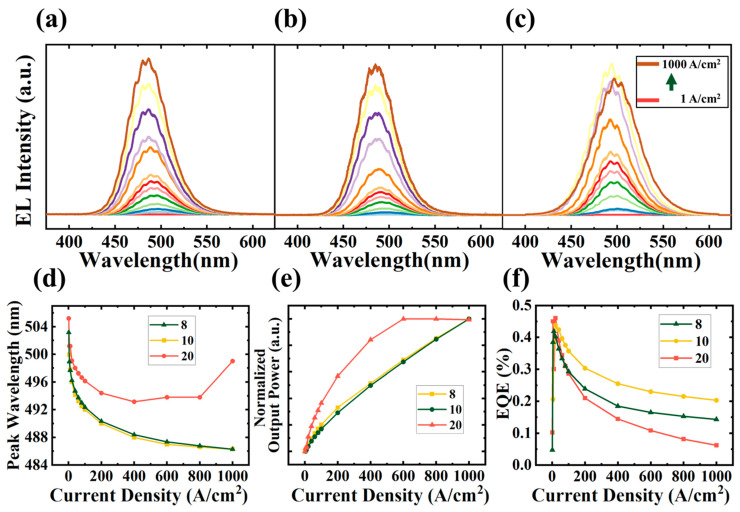
EL spectra of (**a**) 8 µm, (**b**) 10 µm and (**c**) 20 µm InGaN QD micro-LEDs array under injection current densities from 1 A/cm^2^ to 1000 A/cm^2^; the variation of (**d**) EL peak wavelength, (**e**) LOP, and (**f**) EQE with injection current.

Electroluminescence (EL) tests were conducted to measure the emission characteristics of micro-LEDs with side lengths of 8, 10, and 20 µm, as shown in Figure 5a–c. EL measurements were performed with the injection current density from 1 A/cm^2^ to 1000 A/cm^2^. The emission wavelength of 8 µm InGaN QDs micro-LEDs is 503 nm at 1 A/cm^2^. The variations of emission wavelength, light output power (LOP), and external quantum efficiency (EQE) with increasing injection current density were analyzed. It can be found that the InGaN QDs-based green micro-LEDs showed excellent wavelength stability with a shift of less than 17 nm as the injection current density increased from 1 A/cm^2^ to 1000 A/cm^2^. The current density range tested in this work is bigger than the working range of micro-LEDs, but the blue shift of the emission peak is still very slight, which is much smaller than InGaN/GaN MQW micro-LEDs grown on c-plane sapphire [17]. This phenomenon can be attributed to the weaker polarization field in InGaN QD structures [28]. The LOP linearly increased with the increase of injection current for 8 and 10 µm micro-LEDs. However, the 20 µm micro-LED devices exhibited a nonlinear increase in LOP. When the injection current density exceeded 600 A/cm^2^, the LOP especially showed a decreasing trend. Furthermore, the EQE peak values of the 8, 10, and 20 µm micro-LEDs are 0.42% at 10 A/cm^2^, 0.44%, and 0.46% at 20 A/cm^2^, respectively. The peak EQE of the 8 µm micro-LEDs is 91% of the 20 µm devices’ peak EQE. The results indicate that there is no significant decline in the efficiency of QD-based micro-LEDs as the size decreases. However, the efficiency of 20 µm micro-LEDs sharply dropped and became lower than that of the other devices when the injection current density exceeds 60 A/cm^2^.

The efficiency drop of the 20 µm devices is related to the thermal effect of the micro-LEDs [29]. Because sapphire thinning treatment and other effective heat dissipation treatment were not performed during sample packaging, the heat accumulated during the device tests. As the same current density was used in the test, the heat produced by non-radiative recombination and photon reabsorption in the active region of the three sizes micro-LEDs should be consistent. However, to explore the size effect of QD-based micro-LEDs and avoid the interference of electrode shading, no metal electrode was deposited on the surface of the devices so that the current transport layer covered both the surface of p-GaN and the surface of the passivation layer. As shown in Figure 4a, the ITO layer folds at the edge of the contact area marked as A-arrow and the edge of the n-type GaN mesa marked as B-arrow, which would lead to a large resistance positively related to the length of the mesa edge. Therefore, more Joule heat was generated in the ITO layer for larger micro-LEDs under the same injected current density. The wavelength redshift under high current also confirms this conclusion [30], as the redshifted emission wavelength for the 20 µm device originates from a narrower bandgap caused by the higher junction temperature.

The EQE of the InGaN QDs micro-LEDs is still very low. We inferred that it is attributed to the following possible reasons. Firstly, GaN QB layers were grown at low temperatures to protect InGaN QDs, but simultaneously, high-density defects would be introduced and then result in nonradiative recombination. Secondly, as we discussed above, the lack of heat dissipation treatment during the fabrication process of micro-LED would also reduce the luminous efficiency. In addition, because of the smaller electron capture cross section in QDs than that in QWs, the low capture probability has an adverse effect on the micro-LEDs efficiency, so the surface density of InGaN QDs needs to be increased. In a word, the epitaxial structure and fabrication process would be further optimized in future work to improve the performance of InGaN QDs-based micro-LED.

## 4. Conclusions

In summary, self-assembled InGaN QDs material on GaN/sapphire substrate was grown by PA-MBE, and then micro-LEDs based on MQDs structure were obtained. InGaN QDs have a high surface density of over 3.0 × 10^10^ cm^−2^, and good size distribution uniformity. TEM result suggested that abrupt InGaN/GaN interfaces are formed in MQDs structure. In addition, micro-LEDs with square mesa side lengths of 4, 6, 10, and 20 µm were prepared. The EL emission wavelength exhibited a blueshift of 17 nm with the injection current density increasing from 1 A/cm^2^ to 1000 A/cm^2^, and the excellent stability should be contributed to the shielding effect of InGaN QDs on the polarization-related built-in field. At low current density, the EQE of 20, 10, and 8 µm micro-LEDs exhibits a slow and steady decline, indicating that the size effect of QDs micro-LEDs is weak. However, at higher injection currents of over 200 A/cm^2^, 20 µm micro-LEDs show lower EQE and stronger efficiency droop than the other two devices with smaller chip size. We inferred that the anomalous behavior of the 20 µm device was attributed to more heat accumulation in the larger micro-LEDs because of the lack of effective heat dissipation treatment. The redshifted emission wavelength under high current was also caused by this effect.

## Data Availability

The data presented in this study are available within the manuscript.

## References

[1] Parbrook P.J., Corbett B., Han J., Seong T.Y., Amano H. (2021). Micro-Light Emitting Diode: From Chips to Applications. Laser Photonics Rev..

[2] Smith J.M., Ley R., Wong M.S., Baek Y.H., Kang J.H., Kim C.H., Gordon M.J., Nakamura S., Speck J.S., DenBaars S.P. (2020). Comparison of size-dependent characteristics of blue and green InGaN microLEDs down to 1 μm in diameter. Appl. Phys. Lett..

[3] Wu T., Sher C.-W., Lin Y., Lee C.-F., Liang S., Lu Y., Chen S.-W.H., Guo W., Kuo H.-C., Chen Z. (2018). Mini-LED and Micro-LED: Promising Candidates for the Next Generation Display Technology. Appl. Sci..

[4] Choi H.W., Liu C., Gu E., McConnell G., Girkin J.M., Watson I.M., Dawson M.D. (2004). GaN micro-light-emitting diode arrays with monolithically integrated sapphire microlenses. Appl. Phys. Lett..

[5] Huang Y.G., Hsiang E.L., Deng M.Y., Wu S.T. (2020). Mini-LED, Micro-LED and OLED displays: Present status and future perspectives. Light-Sci. Appl..

[6] Sheen M., Ko Y., Kim D.U., Kim J., Byun J.H., Choi Y., Ha J., Yeon K.Y., Kim D., Jung J. (2022). Highly efficient blue InGaN nanoscale light-emitting diodes. Nature.

[7] Chen Z., Yan S.K., Danesh C. (2021). MicroLED technologies and applications: Characteristics, fabrication, progress, and challenges. J. Phys. D Appl. Phys..

[8] Lin J.Y., Jiang H.X. (2020). Development of microLED. Appl. Phys. Lett..

[9] Jin S.X., Li J., Lin J.Y., Jiang H.X. (2000). InGaN/GaN quantum well interconnected microdisk light emitting diodes. Appl. Phys. Lett..

[10] Jin S.X., Li J., Li J.Z., Lin J.Y., Jiang H.X. (2000). GaN microdisk light emitting diodes. Appl. Phys. Lett..

[11] Jain S.C., Willander M., Narayan J., Van Overstraeten R. (2000). III-nitrides: Growth, characterization, and properties. J. Appl. Phys..

[12] Ding K., Avrutin V., Izyumskaya N., Ozgur U., Morkoc H. (2019). Micro-LEDs, a Manufacturability Perspective. Appl. Sci..

[13] Lu S.Q., Li J.C., Huang K., Liu G.Z., Zhou Y.H., Cai D.J., Zhang R., Kang J.Y. (2021). Designs of InGaN Micro-LED Structure for Improving Quantum Efficiency at Low Current Density. Nanoscale Res. Lett..

[14] Itoh H., Watanabe S., Goto M., Yamada N., Misra M., Kim A.Y., Stockman S.A. (2003). Current dependence of in-plane electroluminescence distribution of InxGa1-xN/GaN multiple quantum well light emitting diodes. Jpn. J. Appl. Phys. Part 2 Lett..

[15] Yang F., Xu Y., Li L., Cai X., Li J.J., Tao J.H., Zheng S.N., Cao B., Xu K. (2022). Optical and microstructural characterization of Micro-LED with sidewall treatment. J. Phys. D Appl. Phys..

[16] Huang Y., Tan G., Gou F., Li M.C., Lee S.L., Wu S.T. (2019). Prospects and challenges of mini-LED and micro-LED displays. J. Soc. Inf. Disp..

[17] Zhang M., Bhattacharya P., Guo W. (2010). InGaN/GaN self-organized quantum dot green light emitting diodes with reduced efficiency droop. Appl. Phys. Lett..

[18] Senes M., Smith K.L., Smeeton T.M., Hooper S.E., Heffernan J. (2008). Strong carrier confinement and negligible piezoelectric effect in InGaN/GaN quantum dots. Phys. E-Low-Dimens. Syst. Nanostruct..

[19] Senes M., Smith K.L., Smeeton T.M., Hooper S.E., Heffernan J. (2007). Strong carrier confinement in InxGa1-xN/GaN quantum dots grown by molecular beam epitaxy. Phys. Rev. B.

[20] Raun A., Hu E. (2021). Ultralow threshold blue quantum dot lasers: What’s the true recipe for success?. Nanophotonics.

[21] Lv W.B., Wang L., Wang J.X., Hao Z.B., Luo Y. (2012). InGaN/GaN multilayer quantum dots yellow-green light-emitting diode with optimized GaN barriers. Nanoscale Res. Lett..

[22] Bartel T., Dworzak M., Strassburg M., Hoffmann A., Strittmatter A., Bimberg D. (2004). Recombination dynamics of localized excitons in InGaN quantum dots. Appl. Phys. Lett..

[23] Park I.K., Kwon M.K., Seo S.B., Kim J.Y., Lim J.H., Park S.J. (2007). Ultraviolet light-emitting diodes with self-assembled InGaN quantum dots. Appl. Phys. Lett..

[24] Zhao C., Tang C.W., Lai B., Cheng G., Wang J., Lau K.M. (2020). Low-efficiency-droop InGaN quantum dot light-emitting diodes operating in the “green gap”. Photonics Res..

[25] Wang L., Wang L., Chen C.J., Chen K.C., Hao Z.B., Luo Y., Sun C.Z., Wu M.C., Yu J.D., Han Y.J. (2021). Green InGaN Quantum Dots Breaking through Efficiency and Bandwidth Bottlenecks of Micro-LEDs. Laser Photonics Rev..

[26] Kong X., Albert S., Bengoechea-Encabo A., Sanchez-Garcia M.A., Calleja E., Trampert A. (2015). Lattice pulling effect and strain relaxation in axial (In, Ga)N/GaN nanowire heterostructures grown on GaN-buffered Si(111) substrate. Phys. Status Solidi A–Appl. Mater. Sci..

[27] Hiramatsu K., Kawaguchi Y., Shimizu M., Sawaki N., Zheleva T., Davis R.F., Tsuda H., Taki W., Kuwano N., Oki K. (1997). The composition pulling effect in MOVPE grown InGaN on GaN and AlGaN and its TEM characterization. MRS Internet J. Nitride Semicond. Res..

[28] Jho Y.D., Yahng J.S., Oh E., Kim D.S. (2002). Field-Dependent carrier decay dynamics in strained InxGa1-xN/GaN quantum wells. Phys. Rev. B.

[29] Tian P.F., McKendry J.J.D., Herrnsdorf J., Watson S., Ferreira R., Watson I.M., Gu E.D., Kelly A.E., Dawson M.D. (2014). Temperature-dependent efficiency droop of blue InGaN micro-light emitting diodes. Appl. Phys. Lett..

[30] Lin Y., Gao Y.L., Lu Y.J., Zhu L.H., Zhang Y., Chen Z. (2012). Study of temperature sensitive optical parameters and junction temperature determination of light-emitting diodes. Appl. Phys. Lett..

